# Plant Volatiles Induced by Herbivore Egg Deposition Affect Insects of Different Trophic Levels

**DOI:** 10.1371/journal.pone.0043607

**Published:** 2012-08-17

**Authors:** Nina E. Fatouros, Dani Lucas-Barbosa, Berhane T. Weldegergis, Foteini G. Pashalidou, Joop J. A. van Loon, Marcel Dicke, Jeffrey A. Harvey, Rieta Gols, Martinus E. Huigens

**Affiliations:** 1 Laboratory of Entomology, Research Centre, Wageningen University, Wageningen, The Netherlands; 2 Department of Terrestrial Ecology, Netherlands Institute of Ecology (NIOO-KNAW), Wageningen, The Netherlands; 3 Dutch Butterfly Conservation, Wageningen, The Netherlands; Max Planck Institute for Chemical Ecology, Germany

## Abstract

Plants release volatiles induced by herbivore feeding that may affect the diversity and composition of plant-associated arthropod communities. However, the specificity and role of plant volatiles induced during the early phase of attack, i.e. egg deposition by herbivorous insects, and their consequences on insects of different trophic levels remain poorly explored. In olfactometer and wind tunnel set-ups, we investigated behavioural responses of a specialist cabbage butterfly (*Pieris brassicae*) and two of its parasitic wasps (*Trichogramma brassicae* and *Cotesia glomerata*) to volatiles of a wild crucifer (*Brassica nigra*) induced by oviposition of the specialist butterfly and an additional generalist moth (*Mamestra brassicae*). Gravid butterflies were repelled by volatiles from plants induced by cabbage white butterfly eggs, probably as a means of avoiding competition, whereas both parasitic wasp species were attracted. In contrast, volatiles from plants induced by eggs of the generalist moth did neither repel nor attract any of the tested community members. Analysis of the plant’s volatile metabolomic profile by gas chromatography-mass spectrometry and the structure of the plant-egg interface by scanning electron microscopy confirmed that the plant responds differently to egg deposition by the two lepidopteran species. Our findings imply that prior to actual feeding damage, egg deposition can induce specific plant responses that significantly influence various members of higher trophic levels.

## Introduction

A major challenge in ecology is to understand how phenotypic plasticity of plant traits affects the complexity and dynamics of plant-associated communities. Plants are at the base of food webs, which are defined as networks of feeding connections within an ecological community [1]. Insect herbivores are the most abundant and diverse attackers of plants and induce defensive traits that influence consumers at higher trophic levels [2], [3]. Upon attack by insects, plants emit a blend of volatile organic compounds that affect interactions with organisms belonging to the arthropod community of the plant [4]–[8]. These herbivore-induced plant volatiles (HIPVs) can consist of hundreds of compounds, such as terpenoids, green leaf volatiles and benzenoids and have been shown to act as repellents and/or attractants for herbivores and their natural enemies [4], [5], [8]. HIPVs can provide specific information on the status of the plant to various community members both below- and aboveground, including carnivores, herbivores, pollinators, or neighbouring plants [4], [9]–[12]. Thus, HIPV-mediated effects on different trophic levels imply an extensive effect of plants in structuring associated communities [4], [10], [13].

Although the majority of the about 300,000 described herbivorous insect species [3] deposit their eggs on plant tissues, plant responses elicited by egg deposition, i.e. in the initial phase of herbivore colonization, are still not widely accepted to play a significant role in plant-insect interactions [14]. Yet, an increasing number of studies demonstrates that insect egg deposition can modify (a) the plant’s internal chemistry, with direct consequences for eggs or subsequently feeding herbivores [15]–[19] or (b) the plant’s surface chemistry directly affecting egg survival or indirectly by arresting egg parasitoids, tiny parasitic wasps that kill insect eggs [20]–[28]. Moreover, egg deposition by herbivorous insects has been shown to change plant volatile emission, i.e. oviposition-induced plant volatiles (OIPVs), utilized by parasitoids during host location [29]–[37]. The emission of OIPVs was initially found to require cell damage inflicted by the attacking insects either by wounding caused by the ovipositing female or adult feeding [6], [14], [38]. However, recent studies have indicated that mere egg deposition itself, without wounding, can also enhance or reduce volatile emission with consequences for insect preferences [36], [37], [39].

In the Brassicaceae plant family, egg deposition has been demonstrated to induce resistance responses at the transcriptional level that affect herbivores and parasitoid wasps that attack eggs [27], [40]–[42]. Deposition of eggs by cabbage white butterflies (Pieris spp.) on black mustard plants, *Brassica nigra*, triggers the formation of a necrotic zone at the base of the eggs resembling a hypersensitive response (HR) or programmed cell death, that can lead to egg desiccation and mortality [43]. Moreover, it provokes gene expression changes similar to pathogen-induced HR in Arabidopsis [42]. Egg parasitoids of the genus *Trichogramma* are arrested on the leaf surface of Brussels sprouts plants (*B. oleracea* var. *gemmifera*) when induced by *Pieris brassicae* or *P. rapae* eggs [27], [40], [44]. Here, a butterfly anti-sex pheromone released with the egg-associated secretion was shown to quantitatively change plant surface chemistry [27], [28], most likely epicuticular wax composition, as has been reported for other Brassicaceae [45], [46].

We study oviposition-induced responses in *B. nigra*, an annual wild crucifer native to Europe. This plant species contains high concentrations of glucosinolates as defensive compounds that reduce herbivore growth and survival [47]. Generalist insects like the cabbage moth *Mamestra brassicae* suffer from the toxic breakdown products of glucosinolates, whereas specialists like the larval stages of the Large Cabbage White butterfly *P. brassicae* are adapted to them [48]. Both herbivores lay eggs in clutches on cultivated and wild brassicaceous plant species, such as *B. nigra,* with *M. brassicae* moths having a much larger host plant range than *P. brassicae* butterflies [49], [50]. The generalist wasp *Trichogramma brassicae* is known to parasitize eggs of a wide range of lepidopteran species, including *P. brassicae* and *M. brassicae* [51]. *Cotesia glomerata* is a fairly specialized gregarious endoparasitoid that attacks young instars of *Pieris* spp. in Eurasia.

**Figure 1:**
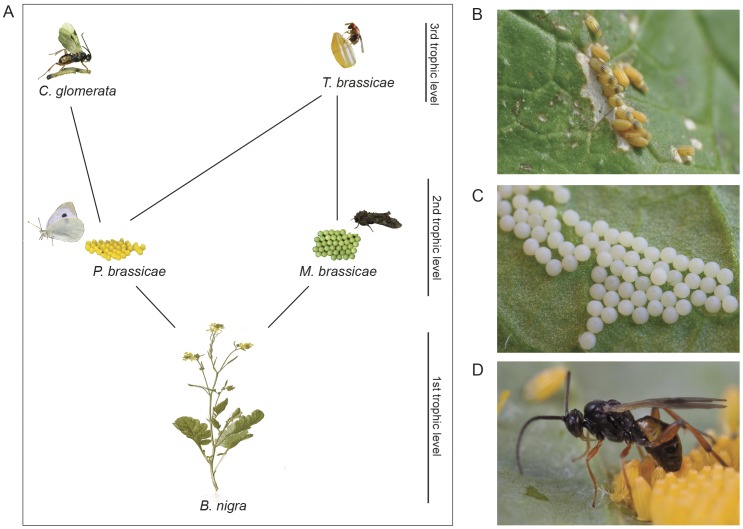
Studied insect community of *B. nigra*. (A) Tritrophic system consisting of the Brassicaceae-specialist *Pieris brassicae* and the generalist moth *Mamestra brassicae* lay eggs in clusters on *B. nigra.* The egg parasitoid *Trichogramma brassicae* attacks eggs of both. The larval parasitoid *Cotesia glomerata* attacks young caterpillar stages of *P. brassicae.* (B) *P. brassicae* clutch on *B. nigra* expressing a strong necrotic zone, i.e. hypersensitive response (HR) (Photo credits: D. Lucas-Barbosa), (C) *M. brassicae* egg clutch on *B. nigra* without necrosis (Photo credits: Nina E. Fatouros, www.bugsinthepicture.com), (D) *C. glomerata* wasp on *P. brassicae* eggs parasitizing a neonate that just hatched (Photo credits: N. E. Fatouros, www.bugsinthepicture.com).

The aim of this study was to investigate a) the effects of egg deposition on plant volatile-mediated interactions with insects at different trophic levels (figure 1) and b) the specificity of the plants’ response to egg deposition by using two different herbivores. We tested the response of the specialist butterfly *P. brassicae* and two parasitoids to volatiles of *B. nigra* plants induced by egg deposition by the specialist butterfly and a generalist moth *M. brassicae*. The behavioural differences were linked to modifications in the composition of volatile blends using gas chromatography coupled with mass spectrometry (GC-MS); cryo-scanning electron microscopy was used to study the bonding region between eggs of the two herbivores and the plant surface.

## Results

### Formation of Necrotic Tissue and Effects on Eggs and Egg Parasitoid

At 24 hours after oviposition (hao) by *P. brassicae*, plants start to express a necrotic zone below the egg clutches (i.e. hypersensitive response, HR+) that sometimes led to egg desiccation or egg drop-off at 72 hao (Figure 1B). All *P. brassicae* egg-infested plants were, therefore, examined for HR and separated from non-HR expressing plants (HR−). On the 10 plants on which eggs were counted, 91% of the eggs did not develop into larvae on HR+ plants, whereas 99% of the eggs hatched on HR− plants (*P*<0.001, 2×2 contingency test using Chi^2^). From these 10 plants, 50% developed HR. In contrast to eggs of *P. brassicae*, eggs of the moth *M. brassicae* did not induce any HR response in *B. nigra*; no necrosis was observed after egg deposition (Figure 1C).

*Trichogramma brassicae* wasps can successfully parasitize and complete their development inside eggs on plants that have expressed HR. The proportion of eggs that was parasitized by *T. brassicae* was not affected by plant phenotype, i.e. occurrence of HR (GLM; χ^2^_1_ = 0.47, *P* = 0.49), but was marginally affected by the age of the eggs, i.e. 24 h or 72 h old (GLM; χ^2^_1_ = 3.91, *P* = 0.053). Older eggs tended to be less parasitized than younger ones. The interaction between plant phenotype and egg age was not significant (GLM; χ^2^_1_ = 0.34, *P* = 0.56). Similarly, there was no effect of plant phenotype on the number of wasp offspring that emerged from parasitized host eggs (GLM; χ^2^_1_ = 0.01, *P* = 0.91), but there was an effect of egg age (GLM; χ^2^_1_ = 6.70, *P* = 0.01). Less offspring emerged from 72 h old eggs than from 24 h old eggs. The effect of egg age was not influenced by the plant’s phenotype (GLM; χ^2^_1_ = 1.15, *P* = 0.28).

### Attraction of Egg Parasitoids

In a dynamic Y-tube olfactometer set-up, the distribution of naïve *T. brassicae* wasps did not differ from 50∶50 in a control test with an uninfested plant in both odour containers (t-test: t_11_ = 0.27, *P* = 0.79). The wasps did not discriminate between clean air and volatiles from uninfested *B. nigra* plants (t-test: t_11_ = −0.10, *P* = 0.92) or clean air and volatiles from plants infested with *P. brassicae* eggs less than 6 hao (t-test: t_9_ = −0.23, *P* = 0.82). However, wasps were attracted to volatiles from *B. nigra* plants infested with *P. brassicae* eggs 24 hao when tested against clean air, irrespective of HR (t-test: HR−: t_9_ = 5.1, *P* = 0.001; HR+: t_9_ = 4.2, *P* = 0.002).

**Figure 2:**
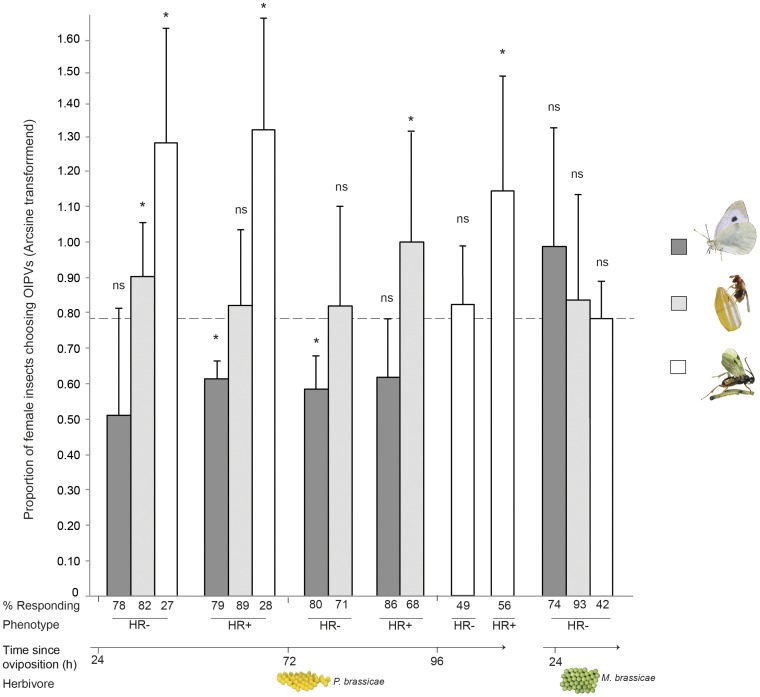
Proportions (±SD) of female insects choosing oviposition-induced plant volatiles (OIPVs) of *B. nigra* plants. Plants were infested with eggs of *P. brassicae* or *M. brassicae*. Columns represent arcsine of the proportion of choice for OIPVs by gravid *P. brassicae* females tested in a flight chamber (dark grey), *T. brassicae* egg parasitoids tested in a Y-tube olfactometer (light grey), and *C. glomerata* larval parasitoids tested in a windtunnel (white). All experiments were conducted in a two-choice situation between plants infested with eggs of different ages (24 h, 72 h, 96 h), and clean plants. The dashed line indicates arcsine (0.5)  =  no preference. Numbers below the columns represent the percentage of female insects making a choice. *P<0.05, one-sample t-test. Each treatment combination was replicated with at least four plant pairs. Different phenotypes: Hypersensitive response (HR), HR−: no necrotic zone observed, HR+: necrotic zone.

The distribution of naive *T. brassicae* wasps choosing egg-induced or non-induced clean plants was marginally affected by the interaction between plant phenotype and egg age (GLM; χ^2^_1_ = 4.14, *P* = 0.059, none of the main effects was significant). Wasps significantly preferred volatiles from plants (HR−) infested with *P. brassicae* eggs (24 hao) when tested against clean control plants (Figure 2; t-test: t_9_ = 2.54, *P* = 0.03). However, they did not discriminate systemically (S) induced volatiles from HR− plants from which leaves with 24 h old eggs had been removed before testing, when tested against uninfested plants (t-test: t_9_ = −0.45, *P* = 0.68). Wasps did not respond to volatiles from HR+ plants 24 hao (Figure 2; t-test: t_9_ = 0.81, *P* = 0.44) or HR− plants 72 hao neither locally (Figure 2; t-test: t_9_ = 0.90, *P* = 0.38) nor systemically (t-test: t_9_ = −1.00, *P* = 0.37) induced. Yet, volatiles from HR+ plants 72 hao significantly attracted wasps locally (Figure 2; t-test: t_11_ = 2.47, *P* = 0.03) and systemically induced (t-test: t_9_ = 2.78, *P* = 0.04).

*T. brassicae* discriminated between volatiles of *M. brassicae* egg-infested *B. nigra* plants 24–36 hao and clean air (T-test: t_11_ = 6.61, *P*<0.001). However, when the same plants were tested against volatiles of uninfested plants, the wasps did not display a preference (Figure 2; t-test: t_11_ = 0.91, *P* = 0.38). At 48–60 hao, the wasps did not discriminate between volatiles emitted by plants infested with moth eggs and clean air (T-test: t_9_ = −1.81, *P* = 0.10).

### Attraction of Larval Parasitoids

In a wind tunnel, the distribution of naive *C. glomerata* female wasps choosing volatiles of egg-induced or non-induced clean plants was only affected by the age of the eggs (GLM; χ^2^_1_ = 8.19, *P* = 0.008) and not by the occurrence of HR (Figure 2; GLM; χ^2^_1_ = 1.99, *P* = 0.17). At 24 hao, *C. glomerata* wasps discriminated between OIVPs and volatiles emitted by non-induced control plants, regardless of the occurrence of HR (Figure 2; t-test: HR−: t_7_ = 4.16, *P* = 0.004; HR+: t_6_ = 4.27, *P* = 0.005), whereas at 96 hao, the preference for OIPVs was less pronounced and was only significant in plants that had developed HR+ (Figure 2; t-test: HR−: t_4_ = 0.72, *P* = 0.51; HR+: t_8_ = 2.93, *P* = 0.019). However, the interaction between plant phenotype and egg age was statistically not significant (GLM; χ^2^_1_<0,01, *P* = 0.95).

*Cotesia glomerata* did not discriminate between volatiles from plants infested with eggs of the non-host *M. brassicae* and uninfested plants 24–36 hao in a wind tunnel set-up (Figure 2; t-test: t_8_ = 0,22, *P* = 0.83). This wasp was not arrested by, and did not show any interest in, *M. brassicae* eggs.

### Avoidance Behaviour of Gravid Butterflies

In a flight chamber set-up, the distribution of gravid *P. brassicae* butterflies first landing on egg-induced or non-induced clean plants was not affected by the age of the eggs (GLM; χ^2^_1_ = 0.08, *P* = 0.77), the occurrence of HR (GLM; χ^2^_1_ = 0.81, *P* = 0.37) nor by the interaction of egg age and plant phenotype (Figure; GLM; χ^2^_1_ = 0.01, *P* = 0.90). Gravid female butterflies tended to first land on plants without eggs, regardless of the age and phenotype of the plants (Figure 2). However, *P. brassicae* butterflies did not discriminate between moth egg-infested plants and control plants 24–36 hao (Figure 2; t-test: t_5_ = −1.26, *P* = 0.27).

### Specificity of OIPV Emission

**Figure 3:**
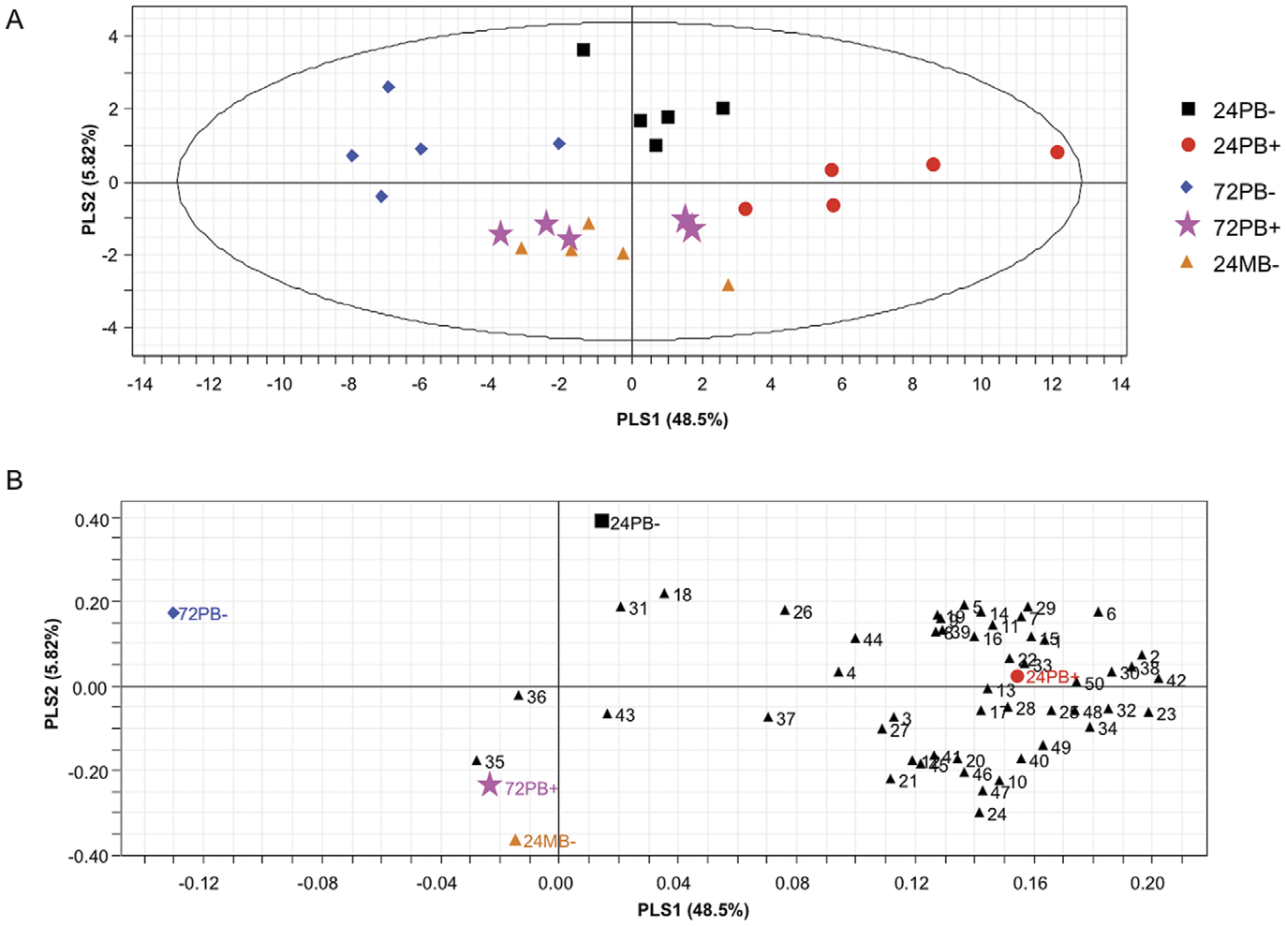
Projection to Latent Structures Discriminant Analysis (PLS-DA) on the volatile compounds emitted by egg-infested *B. nigra.* HR+ (+) or HR− (−) plants were infested by eggs of *Pieris brassicae* (PB) or *Mamestra brassicae* (MB) sampled 24 or 72 h after oviposition. (A) Score plot visualizing the grouping pattern of the samples according to the first two PLS components with the explained variance in brackets. The ellipse defines Hotelling’s T^2^ confidence region (95%). (B) Loading plot of the first two principal components shows the contribution of each of the compounds to the two PLS-DA components. Markers of the 5 different treatments shown in the score plot are given. Hypersensitive response type, (−): no necrotic zone observed, (+): with necrotic zone. Compound numbers: (1) 2-Methylpropanal, (2) 2-methyl-2-Propenal, (3) Ethyl acetate, (4) 2-Methyl-1-propanol, (5) Tetrahydrofuran, (6) 2-Butenal, (7) 3-Methylbutanal, (8) 2-Methylbutanal, (9) 1-Methoxy-2-propanol, (10) 1-Pentene-3-ol, (11) 2-Pentanone, (12) 3-Pentanone, (13) Methylcyclohexane, (14) Pyrazine, (15) 4-Methyl-2-pentanone (16) 1,2-Dimethyldisulfide, (17) (*E*)-2-Pentenal, (18) 2,4-Pentanedione, (19) 4-Methyl-3-pentene-2-one, (20) (*Z*)-3-Hexen-1-ol, (21) Allyl isothiocyanate, (22) α-Pinene, (23) 6-Methyl-2-heptanone, (24) (*Z*)-3-Hexen-1-yl acetate, (25) 3-Carene, (26) (*S*)-Limonene, (27) α-Phellandrene, (28) Methyl-2-ethylhexanoate, (29) (*E*)-**β-Ocimene**, (30) o-Cresol, (31) 2,2,6-Trimethyl-4-methylene-2H-pyran, (32) *p*-Mentha-1,5,8-triene, (33) 2-Phenylacetonitrile, (34) Isopulegon, (35) *p*-Menthan-3-one, (36) **Isomenthone**, (37) Menthol, (38) Undecan-2-one, (39) **7-α-H-Silphiperfol-5-ene**, (40) **Presilphiperfol-7-ene**, (41) **7-β-H-Silphiperfol-5-ene**, (42) 4-(3-cyclohexen-1-yl)-3-Buten-2-one, (43) **Silphiperfol-6-ene**, (44) **α-Funebrene**, (45) Longifolen, (46) 2-Phenylethyl isothiocyanate, (47) Lilial, (48) Guaiazulene, (49) 2-Ethylhexyl salicylate, (50) Cembrene. Significantly increased terpenoids in volatile blends of *P. brassicae* egg-infested plants compared to uninfested plants are in bold (*P<0.05, Mann-Whitney U-test).

The headspace of uninfested *B. nigra* plants was compared with the headspace of *P. brassicae* egg-infested (24 and 72 hao, HR− and HR+) and *M. brassicae* moth egg-infested (24–36 hao, HR−) plants (Table 1). In total, 50 plant-related compounds were detected (present in more than 50% of the replicates of at least the control treatment). A Projection to Latent Structures Discriminant Analysis (PLS-DA) including volatiles of the five different egg treatments of *B. nigra* resulted in a model with two significant principal components (Figure 3A; 2 PLS-DA principal components, R^2^X_cum_ = 0.485, R^2^Y_cum_ = 0.196, Q_2cum_ = 0.159) and separated the five treatments to a large extent. Figure 3B shows the contribution of the emitted compounds to the two principal components. Oviposition by *P. brassicae* significantly suppressed the emission of the majority of compounds in HR− plants at 24 h (Table 1; 34 compounds suppressed, *P*<0.03, sign test) and 72 h (Table 1; 44 compounds suppressed, *P* = 0.001, sign test) compared to uninfested plants. Interestingly, HR+ plants carrying eggs of *P. brassicae* showed an enhanced emission 24 hao compared to uninfested plants (Table 1; 33 compounds enhanced, *P* = 0.05, sign test), whereas at 72 hao the number of compounds showing enhanced emission by HR+ plants was lower (Table 1; 20 compounds enhanced, *P* = 0.20, sign test) and not different to uninfested plants. In HR− plants, the emission rate of 22 compounds was significantly reduced at 72 hao (Table 1). Different forms of the sesquiterpene silphiperfolene (7-α-H-silphiperfol-5-ene, presilphiperfol-7-ene, 7-β-H-silphiperfol-5-ene and silphiperfol-6-ene) were identified for the first time in a *Brassica* species. The total emission of the four silphiperfolenes increased significantly 24 h after *P. brassicae* oviposition (HR−: *P* = 0.05, HR+: *P* = 0.02, Mann-Whitney U-test) as well as the emission of the monoterpene (*E*)-β-ocimene (HR−: *P* = 0.01, HR+: *P* = 0.03, figure 3, table 1). At 72 h after *P. brassicae* oviposition, there was a significant increase in emission of the monoterpene isomenthone (*P* = 0.01) and the sesquiterpene α-funebrene (*P* = 0.02) in HR+ plants (Figure 3, table 1).

**Table 1:** Volatile emission^a^ by *Brassica nigra* HR+ (+) or HR− (−) plants in response to eggs of *Pieris brassicae* (PB) and *Mamestra brassicae* (MB) sampled at 24 or 72 h after oviposition.

	Treatment →	Uninfested	24PB−	24PB+	72PB−	72PB+	24MB−
ID	Compound ↓	(N = 25)	(N = 5)	(N = 5)	(N = 5)	(N = 5)	(N = 5)
**Aliphatic**							
1	2-Methylpropanal	11.56±2.2	7.97±1.5	17.93±4.8	3.57±0.8*	6.63±1.0	4.47±1.3
2	2-Methyl-2-propenal	23.78±4.6	12.19±0.5*	34.63±9.7	5.36±1.3	8.06±1.2	9.26±1.5
3	Ethyl acetate	134.44±25.9	2.92±0.9	21.81±8.0	3.69±1.2	4.29±1.5	12.75±10.4
4	2-Methyl-1-propanol	4.41±0.8	3.82±1.2	6.49±2.8	1.42±0.4	4.18±1.5	1.48±0.4
6	2-Butenal	10.70±2.1	7.28±1.1	17.93±5.9	3.25±0.6*	4.23±0.6	3.85±0.4
7	3-Methylbutanal	12.33±2.4	8.16±1.8	17.44±3.1	5.63±1.8	6.64±1.4	4.45±0.6
8	2-Methylbutanal	8.37±1.6	5.46±2.1	11.08±2.9	4.85±2.4	4.50±1.1	2.89±0.3
9	1-Methoxy-2-propanol	134.94±26.0	192.25±81.7	244.20±78.9	41.31±10.6	87.75±53.5	63.17±14.1
10	1-Penten-3-ol	189.90±36.5	64.13±9.9	396.15±115.0	34.91±15.8*	253.93±134.9	187.68±61.9
11	2-Pentanone	43.81±8.4	47.31±19.5	39.54±11.6	8.06±2.0*	15.20±2.9	18.20±5.5
12	3-Pentanone	53.60±10.3	36.34±22.2	68.65±29.7	8.25±2.0*	78.72±43.8	37.48±12.4
15	4-Methyl-2-pentanone	7.08±1.4	3.92±1.3	6.57±1.9	1.34±0.3	2.23±0.3	2.41±1.1
17	(E)-2-Pentenal	2.53±0.5	1.10±0.3	3.15±1.4	0.31±0.2*	1.28±0.5	1.67±0.7
18	2,4-Pentanedione	55.07±10.6	112.50±89.9	45.09±14.0	2.45±1.2	16.10±7.7	11.76±3.6
19	4-Methyl-3-penten-2-one	68.92±13.3	64.25±35.2	37.59±7.7	8.67±2.5*	23.77±11.2	15.84±5.8
20	(Z)-3-Hexen-1-ol	215.11±41.4	44.56±12.6	179.94±74.5	18.05±6.4*	43.50±18.3	174.64±109.8
23	6-Methyl-2-heptanone	29.31±5.6	17.21±2.5	34.10±4.7	6.63±0.7*	15.83±2.8	16.36±2.8
24	(Z)-3-Hexen-1-yl acetate	573.69±110.4	105.04±18.8	422.09±111.9	45.69±12.3*	171.72±65.4	478.74±173.1
28	Methyl 2-ethylhexanoate	2.81±0.5	1.47±0.7	3.69±1.1	1.29±0.5*	1.98±0.7	0.87±0.5
38	Undecan-2-one	13.61±2.6	11.20±1.0	20.18±4.3	3.94±0.6*	8.54±1.6	7.93±0.8
**Aromatic**							
30	o-Cresol	36.76±7.1	33.26±15.6	49.88±7.9	7.51±1.2*	17.50±2.7	18.05±3.4
33	2-Phenylacetonitrile (benzyl cyanide)	8.91±1.7	4.82±0.6	10.27±4.4	1.91±0.3*	4.39±0.8	3.31±0.9
47	Lilial	18.95±3.6	5.10±0.6	20.95±3.6	2.71±0.5*	20.12±9.6	14.48±8.4
49	2-Ethylhexyl salicylate	5.76±1.1	4.92±3.4	15.31±6.3	1.73±1.4	5.74±3.0	10.85±4.8
**Terpenoids**							
22	α-Pinene	55.24±10.6	22.78±7.1	109.40±44.9	11.74±3.3	12.47±4.4	24.32±12.7
25	3-Carene	48.16±9.3	17.96±8.9	75.99±22.3	5.72±1.5*	13.73±4.7	25.93±13.3
26	(S)-Limonene	68.75±13.2	29.85±11.9	76.41±25.6	14.19±5.4	10.70±3.8	39.98±32.5
27	α -Phellandrene	10.15±2.0	10.19±4.8	11.52±3.0	1.83±0.8*	11.75±7.0	10.99±2.6
29	(E)-β-Ocimene	5.97±1.1	22.69±6.6*	82.28±42.2*	4.55±2.1	5.42±2.0	3.68±2.0
32	p-Mentha-1,5,8-triene	8.91±1.7	3.60±0.9	14.75±3.2	1.63±0.7*	3.01±0.7	4.36±1.3
34	Isopulegon	2.75±0.5	1.20±0.3	4.04±1.0	0.55±0.1*	1.20±0.2	1.42±0.4
35	p-Menthan-3-one	4.62±0.9	1.67±0.7	4.02±1.9	27.09±25.8	23.60±21.9	2.53±1.6
36	Isomenthone	2.50±0.5	2.36±0.3	3.30±0.2	10.65±10.0	10.79±8.8*	1.44±0.7
	**Treatment →**	**Uninfested**	**24PB−**	**24PB+**	**72PB−**	**72PB+**	**24MB−**
**ID**	**Compound ↓**	**(N = 25)**	**(N = 5)**	**(N = 5)**	**(N = 5)**	**(N = 5)**	**(N = 5)**
37	Menthol	25.64±4.9	14.95±4.4	31.32±7.1	47.74±42.9	53.01±40.8	14.64±2.8
39	7-α-H-Silphiperfol-5-ene	88.22±17.0	151.09±61.2	363.17±116.3	–	13.70±13.5	15.88±15.9
40	Presilphiperfol-7-ene	13.36±2.6	15.13±7.6	83.12±34.4	0.06±0.1	6.81±6.8	0.61±0.6
41	7-β-H-Silphiperfol-5-ene	32.16±6.2	46.07±17.4	143.24±55.6	–	4.82±4.8	5.11±5.1
43	Silphiperfol-6-ene	10.09±1.9	17.14±9.2	40.07±15.8	–	2.42±2.4	1.81±1.8
44	α-Funebrene	10.80±2.1	29.44±23.7	8.42±8.4	4.81±2.1	11.26±4.5*	18.37±10.6
45	Longifolen	13.10±2.5	6.66±2.1	17.41±3.1	4.02±1.3	20.06±13.4	6.71±2.2
48	Guaiazulene	10.04±1.9	5.57±1.7	11.40±3.2	2.29±0.7*	3.17±1.4	5.00±1.1
50	Cembrene	16.90±3.3	14.07±8.7	54.27±16.6	1.28±0.8	15.73±3.5	9.78±1.5
**N and/or S containing**							
16	1,2-Dimethyldisulfide	66.36±12.8	39.86±8.1	420.67±341.7	33.40±20.2	19.96±2.1	22.90±5,2*
21	Allyl isothiocyanate	393.18±75.7	63.65±38.0	782.07±391.7	53.90±20.8*	147.79±50.1	641.32±565.5
46	2-Phenylethyl isothiocyanate	13.86±2.7	3.06±1.0	20.23±7.8	1.06±0.4*	10.81±2.9	3.92±0.9*
**Cyclic/Heterocyclic**							
5	Tetrahydrofuran	2.49±0.5	1.96±0.2	4.66±1.3	1.37±0.4	1.07±0.2	1.65±0.8
13	Methylcyclohexane	11.56±2.2	5.20±0.8	19.64±8.4	3.79±1.7	4.08±1.2	6.73±2.3
14	Pyrazine	9.15±1.8	7.32±2.4	12.03±2.5	3.58±1.4	3.28±1.0	3.38±0.5*
31	2,2,6-Trimethyl-4-methylene-2H-pyran	21.35±4.1	55.11±47.3	12.36±2.9	3.43±1.9	6.20±2.3	9.69±5.5
42	4-(3-Cyclohexen-1-yl)-3-buten-2-one	4.65±0.9	5.58±1.8	9.73±4.0	1.51±0.8	1.19±0.7	4.60±1.2

a Volatile emissions are given in mean peak area ± SEM/g fresh weight of foliage divided by 10^5^ with the number of replicates between brackets.

* values with asterisk indicate significant differences in emission quantities between oviposition-induced *B. nigra* and uninfested control for each treatment (Mann-Whitney U-test).

Oviposition by *M. brassicae* moths significantly suppressed the emission of the majority of compounds compared to uninfested plants (Table 1; 43 compounds suppressed, *P*<0.001, sign test). The emission of terpenes did not change after *M. brassicae* moth oviposition; however, there was a significant reduction in the emission of three compounds, *i.e.* 1,2-dimethyldisulfide (*P* = 0.02), and 2-phenylethyl isothiocyanate and pyrazine (Table 1; both: *P* = 0.05).

### Specificity of Changes in Plant Surface Structure

**Figure 4:**
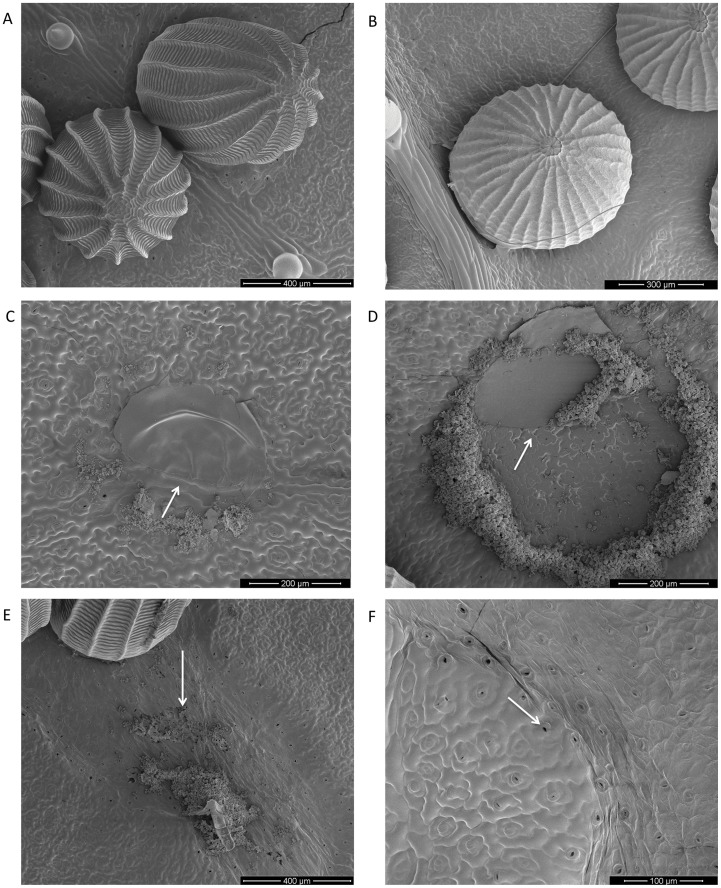
Cryo-SEM micrographs of *B. nigra* leaf surfaces and adhering herbivore eggs and egg – leaf contact regions. (A–E) abaxial site of *B. nigra* leaves. (A) Eggs of *P. brassicae* 72 hao with surrounding leaf surface of HR+ *B. nigra* and trichomes. (B) Eggs of *M. brassicae* (48–60 h old) with surrounding leaf surface of HR− *B. nigra*. (C) Contact region after *P. brassicae* egg removal (72 hao) on HR− *B. nigra* consisting of accessory reproductive gland (ARG) secretion functioning as cement (arrow). (D) Contact region after *M. brassicae* egg removal consisting of a part of ARG cement and healthy leaf cells with open stomata (arrow). (E) Necrotic zone on HR+ *B. nigra* leaf induced by *P. brassicae* eggs 72 hao, with some eggs removed. ARG-derived cement is removed together with parts of the cell layer (arrow) and open stomata at the border between dead and living cells. (F) Adaxial site of necrotic zone of HR+ *B. nigra* with open stomata (arrow) between healthy cells and necrotic zone on right side. Hypersensitive response type, HR−: no necrotic zone observed, HR+: with necrotic zone. Scale bars are given in lower right corners.

*Pieris brassicae* butterflies and *M. brassicae* moths carefully deposit their eggs on *B. nigra* plants without any visible damage to the surface in the vicinity of the eggs (Figure 4A–B). Egg cement is produced by the accessory reproductive gland and attaches the eggs of *P. brassicae* and *M. brassicae* to the substrate (Figure 4C–D). After freezing, we observed that moth eggs detached more easily from the plant surface than eggs of *P. brassicae*. Egg secretion of *P. brassicae* partly peeled off after egg removal, covering the surface of HR− *B. nigra* with a thick layer. Epidermal cell morphology and stomata are not visible under the egg cement of *P. brassicae* (Figure 4C). Part of the thin egg secretion of *M. brassicae* moths seems to be peeled off after egg removal or is missing. The cell layer below the egg cement seems to be intact and stomata are half opened (Figure 4D). In HR+ plants, necrotic tissue develops within 72 h and is only induced by *P. brassicae* eggs. Here, egg removal leads to detachment of surface layers of dead cells together with the egg secretion (Figure 4E). Interestingly, stomata are open adjacent to cells at the boundary of the necrotic zone, supposedly in the programmed cell death phase (Figure 4F).

## Discussion

Our study revealed that plant volatiles induced in the early phase of colonization by insect herbivores, before actual feeding starts, mediate interactions between a range of insect community members at different trophic levels: egg and larval parasitoids are attracted and the specialist herbivore prefers plants that are free of eggs. Moreover, we show that the plant and associated insects respond differently to egg deposition by two herbivores, the specialist butterfly *P. brassicae* and the generalist moth *M. brassicae*. Oviposition by the abundant specialist pine sawflies *Diprion pini* and *Neodiprion sertifer* on *Pinus sylvestris* induced the emission of pine volatiles that attracted the specialized egg parasitoid *Chrysonotomyia ruforum,* whereas eggs of the less abundant pine sawfly *Gilpinia pallida* did not induce such a response [32].

OIPVs may provide an effective defence against the attacking herbivore: egg parasitoids kill a certain proportion of the host eggs; and the remaining proportion of eggs yields caterpillars that may be attacked by larval parasitoids. Gravid butterfly females avoid plants infested by eggs of conspecifics by using OIPVs. Herbivore-induced plant volatiles have been shown to mediate interactions with other herbivores [52], [53], but to the best of our knowledge this has not been reported for OIPVs. In combination with limited feeding damage, odours of plants carrying few eggs were shown to be avoided by gravid elm leaf beetles [54]. Recently, Bruce et al. [39] showed that the spotted stemborer *Chilo partellus* avoided oviposition on egg-induced African forage grass (*Brachiaria brizantha*), but the role played by visual and contact cues here was not determined.

Furthermore, we here show that parasitism of *P. brassicae* eggs by *T. brassicae* wasps on HR+ and HR− plants was equally successful, which means that there is no conflict between the induced hypersensitive response and the performance and attraction of the egg parasitoid. While eggs of *P. brassicae* induce HR in about 50% of the observed *B. nigra* plants, eggs of *M. brassicae* moths did not induce the formation of necrotic tissue. Eggs of cabbage white butterflies, moths and beetles have been shown to induce the formation of necrotic tissue leading to increased egg mortality on different plant species, including the wild crucifer *Sinapis arvensis* (F. G. Pashalidou, personal observations), potato [22] and *Physalis* plants [20]. A whole-genome transcriptomic study with *Arabidopsis* confirmed that oviposition by *Pieris* sp. triggers a defensive response with strong similarities to microbial-induced HR, i.e. up-regulation of pathogenesis-related genes, callose accumulation, and production of reactive oxygen species [42].

For successful settlement of an herbivorous insect, it is crucial that eggs are deposited with a proper adherence. How firmly eggs are attached to the leaf surface may affect different cells that are able to perceive information about when an egg has been laid [38]. Epicuticular waxes lead to hydrophobic surfaces which can prevent insect egg attachment [55]. *Pieris brassicae* is specialized to deposit eggs on leaf surfaces typical of species in the Brassicaceae plant family. A water-soluble yellow phenolic compound of the egg cement probably moistens the surface and increases the strength of adhesion [56]. Generalist herbivores such as *M. brassicae* moths are expected to be less adapted to certain plant surfaces; their eggs seem less firmly attached to the hydrophobic *B. nigra* surface. From the plant’s plants perspective, we expect less selection pressure on *B. nigra* to respond to eggs of the less abundant generalist *M. brassicae* because their larvae perform poorly on wild crucifers containing high levels of secondary compounds, i.e., glucosinolates [57], and tend to leave such plants quickly after emergence (J.A. Harvey and F.G. Pashalidou, personal observations). Egg deposition by *Spodoptera frugiperda* moths was shown to suppress HIPV emission in maize while eggs were in close contact with the plant cuticle, accompanied with accessory gland secretion. A possible explanation for the HIPV suppression was that the *S. frugiperda* egg masses are dense and cover parts of the photosynthetic tissue, thus inhibiting the volatile emission [58]. Indeed, oviposition was shown to reduce photosynthesis, which several workers have suggested is caused by the coverage of photosynthetic tissue and/or physiological mechanisms, i.e. reduced CO_2_ diffusion in the mesophyll or water deficiency [59], [60]. Unlike with *M. brassicae* eggs, stomata were closed underneath the eggs of *P. brassicae* and gas/water exchange probably inhibited in *B. nigra* (Figure 4C–D). However, although eggs of *M. brassicae* are covering a slightly larger part of the leaf surface than *P. brassicae* eggs (Figure 1B–C), it is unlikely that this would lead to significant differences in the volatile emission demonstrated here. The observed attraction to volatiles from *M. brassicae* egg-infested plants by *T. brassicae* at 24 hao when tested against clean air is probably caused by the moths’ sex pheromone adsorbed to the plant surface, previously shown to attract *Trichogramma* wasps 24 h after release [61].

Chemical analysis of volatile blends revealed reduced emissions for the majority of chemical compounds in the plant treatments with eggs. Usually, insect herbivory leads to an increase in the emission of plant volatiles that attract carnivorous natural enemies [9], [62]. A reduced emission induced by egg deposition has recently been demonstrated in other plant species as well [39], [58]. Only HR+ plants 24 h after *P. brassicae* oviposition showed an increased emission, probably due to the initiation of necrosis. A significant induction of some terpenoids might contribute to the specificity of *P. brassicae* egg-induced volatile blends that are attractive or repellent to the tested insects. For example, the emission of (*E*)-β-ocimene was enhanced in *B. nigra* 24 h after *P. brassicae* oviposition. This monoterpene has been shown to be highly inducible by herbivory [5], [8], [63]. In their study on African grass, Bruce et al. [39] demonstrated similar effects: oviposition by *C. partellus* reduces the plant volatile emission of the main compound, the green-leaf volatile (*Z*)-3-hexenyl acetate, and increases the emission of minor compounds, i.e. terpenoids, causing an increased attraction of the larval parasitoid *Cotesia sesamiae*. The same wasps were shown to be attracted also to synthetic terpenoids [36].

Whether the attraction to OIPVs by different parasitoid species and avoidance by herbivores is adaptive for the plant and eventually leads to enhanced plant fitness remains to be proven. Kessler & Heil [64] argued that HIPV-mediated reduction in herbivory may not result in increased plant fitness because most natural enemies do not immediately kill the herbivore, plants have a high tolerance to herbivory, and HIPV are part of a network with many more functions. However, the results of our study suggest a benefit for *B. nigra* resulting from the release of OIPVs. Idiobiont parasitoids that immediately kill the host such as *Trichogramma* spp. are likely to have a greater impact on plant fitness than parasitism by koinobiont parasitoids, which allow the parasitized host to continue to feed. *Trichogramma* wasps have been demonstrated to be significant mortality factors for eggs of *Pieris* species in the field. In an on-going field survey of a Dutch *B. nigra* population, 30–40% of the collected *Pieris* eggs were found to be parasitized by *Trichogramma* spp. (N.E. Fatouros, unpublished data) [65]. Feeding by *Pieris* caterpillars can have detrimental effects on flowering brassicaceous plant species: *P. brassicae* caterpillars have been shown to move to and preferentially feed on the flowers of *B. nigra* plants a few days after hatching [66], [67]. Moreover, the multifunctional effects of OIPVs released by *B. nigra* on different members of the insect community demonstrated here is beneficial to the plant: direct (egg-killing HR and avoidance by female butterflies) and indirect (parasitoid attraction) defence traits against *Pieris* butterflies work in concert which seems to lead to high *Pieris* egg mortality rates under natural conditions (N.E. Fatouros, unpublished data) [65].

Both parasitoid species studied here discriminated between volatiles induced by eggs of their host *P. brassicae* and uninfested *B. nigra* plants, but not between plant volatiles induced by eggs of the moth *M. brassicae* and uninfested *B. nigra*. For *C. glomerata*, *M. brassicae* cannot serve as a host and, therefore, it may be adaptive for the wasps to discriminate between host- and non-host induced plant volatile blends. Studies on brassicaceous plant species demonstrated that naïve *C. glomerata* failed to discriminate between HIPV blends from host and non-host insects [68] or from different host instars [69]. Volatiles emitted by *B. oleracea* plants damaged by different herbivores were shown to be very similar [9], [12]. So far, a single study revealed that parasitoids can innately use HIPV blends to discriminate between host and non-host herbivores [70]. Approaching host-infested plants in an early stage of host development might help *Cotesia* wasps to find host patches and avoid to fly to patches of older host larvae, which are unsuitable for development [71]. Recent studies on different maize varieties and a grass species induced by eggs of the stemborer moth *C. partellus* confirmed an attraction to OIPVs by a larval parasitoid [36], [37], [39].

Our data reveal an effect of an induced plant response on members of the insect community at different trophic levels during the pre-feeding phase of herbivore colonization. The synergistic effect of OIPVs attracting different parasitoid species and causing avoidance by herbivores might lead to an effective reduction of fitness loss caused by a common insect herbivore of brassicaceous plant species. Our findings thus suggest that studies on plant defences induced by herbivores should consider the first phase of herbivore attack before feeding damage has occurred, because of it significant impact on multi-trophic interactions. As a follow-up, we are currently investigating the role of OIPVs under natural conditions to fully understand the consequences of plant-mediated effects of insect egg deposition for the structure and dynamics of arthropod communities.

## Materials and Methods

### Plants and Insects

Black mustard plants (*B. nigra* L.) were grown in a greenhouse (18±5°C, 50–70% r.h., L16:D8). Seeds originated from the Centre for Genetic Resources (CGN, Wageningen, The Netherlands). This accession (feral population, collected in 1975 from the Peloponesus, Greece) had been multiplied by exposing them to pollinators in a common garden experiment in the surroundings of Wageningen, The Netherlands [66]. Plants of 3 to 5 weeks old were used in the experiments. All used insects were collected in the surroundings of Wageningen, The Netherlands. No specific permits were required for their collection. The collection sites were not privately owned or protected in any way and field samplings did not involve endangered or protected species. Mated females of *P. brassicae* (Lepidoptera: Pieridae) were obtained by pairing a virgin male and a virgin female butterfly one day after eclosion. Two days after mating, *P. brassicae* females were used in the experiments. Female *M. brassicae* L. (Lepidoptera: Noctuidae) moths were placed together with a *B. nigra* plant in a cage to allow egg deposition. Both herbivorous insects were reared on Brussels sprouts plants (*B. oleracea* var. *gemmifera* cv. Cyrus) in a climate room (21±1°C, 50–70% rh, L16:D8). *Trichogramma brassicae* Bezdenko (Hymenoptera: Trichogrammatidae) was reared in eggs of the moth *Ephestia kuehniella* (Koppert, Berkel en Rodenrijs, The Netherlands) in a climate chamber (25±1°C, 50–70% rh, L16:D8). Only mated, 2–5 days old, wasps were used in the experiments. The larval parasitoid *Cotesia glomerata* L. (Hymenoptera: Braconidae) was reared in *P. brassicae* caterpillars, feeding on Brussels sprouts plants in a greenhouse (see above). Only mated, 2–8 days old female wasps were used in the experiments. None of the wasps used in the experiments have had previous contact with any plant material or host residues and the wasps are referred to as naïve.

### Plant Treatments

For bioassays with egg-infested plants, test plants were placed into a cage with more than 100 *P. brassicae* adults (female: male ratio 1∶1) to allow deposition of eggs onto the plants. Plants were exposed for no more than 15 min to the butterflies, to obtain 2–3 egg clutches. After this exposure time, egg-infested plants were tested immediately or kept in a greenhouse compartment (21±2°C, 70% r.h., L16:D8) either overnight (24 h) or for 48 to 72 h following egg deposition. Thus, the duration of induction in response to egg deposition was less than 6 to 96 h. Around 120 hao eggs started to hatch. Plants used as controls were kept under the same conditions as the treated plants but had not been in contact with *P. brassicae* or any other insect. To test for a systemic induction of volatiles, butterflies were allowed to oviposit on the lower leaves and the upper leaves were covered with a mesh bag that prevented oviposition. Bags were removed afterwards. Prior to bioassays, leaves with eggs were removed. Leaves at similar stem positions were removed from control plants. Plants with 2–5 *M. brassicae* egg clutches were obtained by exposing plants to *M. brassicae* females during the scotophase. These plants were incubated for an additional one or two days in a greenhouse compartment. Thus, eggs were 24–36 or 48–60 h old when the plants were used in the bioassay.

### Egg-induced Necrosis

All egg-induced plants were checked for the formation of necrotic tissue, referred to as hypersensitive response (HR) 24 h and 72 h after oviposition. The strength of HR was recorded and the plants were categorized into HR− (no necrotic zone observed) and HR+ (necrotic zone +/− eggs fallen off). Plants were kept under greenhouse conditions (22±2°C, 70% r.h., L16: D8). From 10 plants, the number of plants with necrosis was noted and the number of eggs was counted directly after oviposition and after 5 days.

### Egg Parasitoid Performance

To investigate whether the performance of *T. brassicae* in eggs deposited on plants that respond with HR is affected, we infested at least five different plants with *P. brassicae* eggs and offered them 24 h or 72 h after oviposition to *T. brassicae*. Previous research showed that *Trichogramma* wasps were able to parasitize *P. brassicae* eggs 0–72 hao [26]. An egg-carrying leaf of an HR+ or HR− plant was excised and a piece of it carrying 8 eggs (about 2 cm^2^) was offered to a 2–3 days old inexperienced female *T. brassicae* wasp in a glass tube. After 48 h, wasps as well as hatching eggs were discarded. Successful parasitism was checked after 7 days and emerging offspring were counted 12 days after oviposition. In total, 15 female wasps were tested for each treatment.

### Dynamic Y-tube Olfactometer

**Figure 5:**
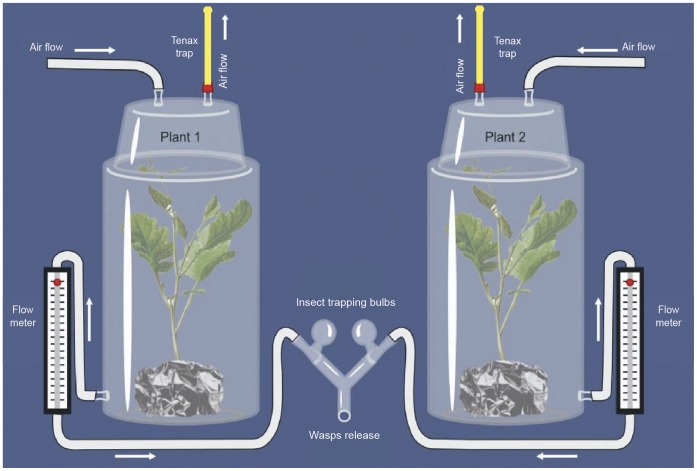
Overview of the Y-tube olfactometer with simultaneous volatile trapping. Wasps were released in groups and collected in insect trapping collection bulbs. Volatiles were trapped simultaneously or after a bioassay with Tenax TA tubes. Illustration credits: I. Figueroa.

Bioassays with *T. brassicae* wasps were conducted in a dynamic airflow Y-tube olfactometer, a modified version of the six-arm olfactometer developed by Turlings et al. [72] (Figure 5). This olfactometer was adapted for small wasps like *Trichogramma sp.*; wasps were released in groups collected in so-called insect trapping bulbs (Figure 5). Pressurized air was filtered through activated charcoal and approximately 150 mg of Tenax-TA 25/30 mesh (Grace-Alltech) before entering the system. Subsequently, air was humidified by passing through a bottle containing 50 mL of tap water. A flow meter-controlled (Brooks Instrument B.V., Veenendaal, NL) airflow of 400 mL min^−1^ was admitted into the system. The airflow was split into two and each subflow was led into a glass container (45 mm high, 200 mm diameter) holding an odour source through an inlet situated on the lid. These containers were sealed airtight using a Viton O-ring and a metal clamp. Air from each odour container was subsequently led into one of the arms of a glass Y-tube olfactometer (stem 9 cm, arms 8 cm, ID 1 cm). All glass parts were connected using Teflon tubing. The airflow was set at 100 mL min^−1^ in each arm using flow meters. Experiments were carried out between 10∶00 and 16∶00 h in the laboratory at 21±2°C using a T5-growth light with a spectrum that is close to sunlight. Light bulbs (4×24 W) were situated above the olfactometer and the containers with the odour sources. Just before placing a plant in the odour containers, the pot of the plant was removed and the roots and soil were tightly covered with aluminium foil.

Ten adult females of *T. brassicae* were released simultaneously and their preference for one of the two odour sources was recorded. Wasps, which were attracted to light, were trapped in two round trapping bulbs connected to the Y-tube near the end of each arm. After 30 minutes, the wasps collected in each of the trapping bulbs were counted. When a wasp did not make a choice within 30 minutes, it was recorded as a “no response” and excluded from the statistical analysis. In total, 100–120 wasps were tested with 5–6 different plants per odour source combination with two replicates per experimental day. Each wasp was used only once. To exclude any bias, the position of the odour sources was exchanged after every trial.

### Flight Chamber Experiments

Butterfly odour preferences were tested in a two-choice situation in a flight tent as described by Gols et al. [70]. A female butterfly was released 80 cm away from the uninfested and egg-infested plant, which were placed 55 cm apart. Eggs were removed just prior to testing. After releasing the butterfly, first landing and oviposition was recorded, after which the observation was ended and the female and her eggs were removed immediately. Females that did not respond within 15 min were recorded as “no response” and excluded from the analysis. Plants were switched after 3 consecutive butterfly observations with a total of max. 10 responding females per set of plants. Plants had been infested with eggs for either 24 h or 72 h (for *P. brassicae*) or 24–36 h (for *M. brassicae*). Per treatment, 43–85 butterflies were tested and 4–6 sets of plants were used.

### Wind Tunnel Experiments

Attraction of *C. glomerata* wasps was conducted in a wind tunnel set-up described in detail by Geervliet et al. [73]. Females were released individually 70 cm down-wind from the two plants, one egg-infested plant and an uninfested control plant. The plant on which the female landed for the first time within 10 min following release was recorded. Non-responding wasps, i.e. those females that did not land within 10 min were counted but excluded from the statistical analysis. Each wasp was used only once. During bioassays, plants were switched after every second wasp tested. The number of tested wasps ranged between 60 and 105 wasps per treatment, tested on at least 5 different days with 5 new sets of plants.

### Headspace Collection of Volatiles

When testing the response of *T. brassicae* wasps to *B. nigra* volatiles using the Y-tube olfactometer (see above), we simultaneously or afterwards collected volatiles from the headspace of the same plant (s) (Figure 5). Volatiles were collected by sucking air with odours out of a glass jar at a rate of 80 mL min^−1^ for 4 h through a stainless steel cartridge filled with 200 mg Tenax TA (20/35 mesh; CAMSCO, USA). A pump (PAS-500 SPECTREX, US) was directly connected to the cartridge steel tube with Tenax TA onto the outlet for sucking the air out of the glass jar. In total, 5 plant pairs (control vs. treatment) were sampled per treatment on 5 different days. Aerial parts of the plant were weighed after volatile collection (balance Mettler-Toledo B.V., NL).

### Chemical Analysis

Thermo Trace Ultra gas chromatography (GC) coupled with Thermo Trace DSQ quadruple mass spectrometer (MS) (Thermo Fisher Scientific Waltham, USA) were used for separation and detection of plant volatiles. Prior to release of volatiles, Tenax TA cartridges were dry-purged under a stream of nitrogen at 20 ml min^−1^ for 10 min at ambient temperature in order to remove moisture. The collected volatiles were released thermally from the Tenax TA in an Ultra 50∶50 thermal desorption unit (Markes, Llantrisant, UK) at 250°C for 10 min under a helium flow of 20 ml^−1^ while re-collecting the volatiles in a thermally cooled universal solvent trap at 10°C using Unity (Markes, Llantrisant, UK). Once the desorption process was completed, the cold trap was heated fast at 40°C s^−1^ to 280°C and was kept for 5 min at 280°C, while the volatiles were released to a ZB-5MSi capillary column with dimensions 30 m L×0.25 mm I.D.×1.00 µm F.T. (Phenomenex, Torrance, CA, USA), in a split mode at a split ratio of 5∶1 for further separation. The GC oven was operated at an initial temperature of 40°C and was immediately raised at 8°C min^−1^ to 280°C and held there for 4 min under a helium flow of 1 mL min^−1^ in constant flow mode. The DSQ MS was operated in scan mode with a mass range of 35–350 amu at 5.38 scans s^−1^ and ionization was performed in EI mode at 70 eV. MS transfer line and ion source were set at 275 and 250°C, respectively.

Identification of compounds was based on comparison of mass spectra with those in the NIST 2005 and Wageningen Mass Spectral Database of Natural Products MS libraries. Experimentally obtained linear retention indices (LRI) were also used as additional criterion for confirming the identity of compounds. Relative quantitation (peak areas of individual compounds) was carried out using a single (target) ion, in selected ion monitoring (SIM) mode. These individual peak areas of each compound were corrected for the aerial fresh weight of each plant sample and were used for further characterization of the different plant groups using statistical analysis.

### Cryo-SEM Imaging

Abaxial and adaxial sites of fresh leaves with *P. brassicae* or *M. brassicae* eggs were fresh-frozen and analysed by field emission scanning microscopy (Magellan 400, FEI, Eindhoven, the Netherlands). Leaves were glued on a brass Leica sample holder by carbon glue (Leit- C, Neubauer Chemicalien, Germany), flash-frozen in liquid nitrogen and simultaneously fitted in a cryo-sample loading system (VCT 100). The Leica sample holder was transferred to a non-dedicated cryo-preparation system (MED 020/VCT 100, Leica, Vienna, Austria) onto a sample stage at −93°C. In this cryo-preparation chamber samples were freeze dried for 2 minutes at −93°C at 1.3×10^−6^ millibar to remove water vapor contamination from the surface of the sample. The sample was sputter-coated with a layer of 15 nm Tungsten at the same temperature. The samples were transferred in high vacuum into a field emission scanning microscope (Magellan 400, FEI, Eindhoven, the Netherlands) on the sample stage at −122°C at 4×10^−7^ millibar. The analysis was performed with SE at 1 and 2 kV, 13 pA. All images were recorded digitally.

The interface between the *B. nigra* surface and (1) 72 h old *P. brassicae* eggs (HR− and HR+) and (2) 48–60 h old *M. brassicae* eggs (HR−) was comparatively studied (a) in contact and (b) with eggs detached after being frozen.

### Statistical Analysis

To analyse whether the distribution of behavioural choices of butterflies and wasps was affected by egg age and plant phenotype, a generalized linear model (GLM) with a logit link function and a binomial distribution for errors was used. The response of a number of animals tested with one set of plants served as experimental unit in the analyses. For each phenotype - -egg age - -animal species-combination at least five newly prepared plant combinations were used. The responses were analysed separately for the three animal species. When overdispersion was detected in the variance parameter, we corrected for this by allowing the variance functions of the binomial distribution to have a multiplicative overdispersion factor by dividing the square root of the deviance of the model by the degrees of freedom.

To determine whether there was a preference for an odour source within a treatment combination, we used one sample t-test on the proportion of wasps preferring egg induced volatiles in each replicate. Data were arcsine-transformed and tested against arcsine (0.5), i.e. no preference for either odour source. Non-responding wasp were excluded from the analyses (both GLM and t-tests).

Percentages of *P. brassicae* eggs hatching on HR+ and HR− plants were compared with a chi-square test using a 2×2 contingency table. Performance of *T. brassicae* on HR+/− plants in relation to egg age was also analysed using a GLM. The proportions of *P. brassicae* eggs that were parasitized by *T. brassicae* were analysed using the same GLM approach as for the behavioural responses. The offspring numbers were compared with a logarithm link function and a Poisson distribution for the errors.

Volatile compounds, measured as peak area divided by the fresh weight of a plant’s foliage were analysed using the software program SIMCA P+12.0 (Umetrics AB, Umeå, Sweden). A PLS-DA was used to determine whether samples belonging to specific groups (here treatments) could be separated based on quantitative differences in volatile emissions [74]. A Y-data matrix of dummy variables was included, which assigns a sample to its respective class. The PLS-DA extension of the SIMCA P+12.0 program used for this analysis approximates the point ‘swarm’ in X (matrix with volatile compounds) and Y in PLS components in such a way that maximum covariation between the components in X and Y is achieved. The results of the analysis were visualised in score plots, which reveal the sample structure according to the model components, and loading plots, which display the contribution of the volatile emission to these components, as well as the relationships among the variables. PLS-DAs were performed on full data sets including all volatile compounds and on restricted data sets containing compounds of which the VIP (Variable Importance in the Projection) values were greater than 1. Data were log-transformed, mean-centred, and scaled to unit variance before they were subjected to the analysis.

A Mann-Whitney-U-test was used to test differences in peak area per compound between treated and control plants. A sign test was used to determine whether the number of compounds emitted in larger or smaller amounts differed from a 50∶50 distribution.
